# Rooibos herbal tea: An optimal cup and its consumers

**DOI:** 10.4102/hsag.v24i0.1090

**Published:** 2019-02-21

**Authors:** Hannelise Piek, Irma Venter, Fanie Rautenbach, Jeanine L. Marnewick

**Affiliations:** 1 Department of Biotechnology and Consumer Science, Cape Peninsula University of Technology, South Africa; 2 Department of Biomedical Sciences, Cape Peninsula University of Technology, South Africa; 3 Oxidative Stress Research Centre, Institute of Biomedical and Microbial Biotechnology, Cape Peninsula University of Technology, South Africa

## Abstract

**Background:**

Rooibos types and forms and how prepared and flavoured influence the total polyphenol content and total antioxidant capacity (TAC).

**Aim:**

To denote an optimal rooibos cup as having the highest total polyphenol content and TAC, considering the different types, forms, preparation methods and flavourings and amounts (Phase 1), and determine the demographic, lifestyle and rooibos consumption characteristics of adult rooibos consumers, and the association of these characteristics with drinking the optimal cup (Phase 2).

**Setting:**

Assays: Oxidative Stress Research Centre, Cape Peninsula University of Technology; Consumer survey: George area, South Africa.

**Method:**

Phase 1 entailed determining the total polyphenol content (Folin–Ciocalteau method) and TAC (Trolox equivalent antioxidant capacity and ferric-reducing antioxidant power assay) of the prepared rooibos samples. For Phase 2, a developed, pilot tested questionnaire was used to profile adult rooibos consumers.

**Results:**

Phase 1: the following samples delivered higher total polyphenol content and TAC: green (type), green leaves and powdered extract (forms), and sample steeped for 10 min or longer (preparation method). The identified optimal cup was sample steeped for 10 min or longer. Phase 2: a total of 308 respondents completed the questionnaire. Few consumed more than one rooibos cup per day (25.3%; *n* = 78) and the optimal cup (15.9%; *n* = 49). These latter respondents comprised those who steeped rooibos in a teapot (not a cup or mug) (*p* < 0.05).

**Conclusions:**

The optimal cup was identified as sample steeped for 10 min or longer. The rooibos consumers did not consume it sufficiently, nor steeped it long enough.

## Introduction

### Research background

Limited research has been conducted on the preparation of rooibos (*Aspalathus linearis*) herbal tea as a beverage to deliver a maximal polyphenolic content and total antioxidant capacity (TAC) or to obtain an optimal cup of rooibos herbal tea. Beverage consumption, in particular of tea, makes a large contribution to daily polyphenolic intake (Manach et al. 2004) and dietary TAC (Louwrens, Rautenbach & Venter 2009). Rooibos herbal tea is an excellent source of polyphenolic flavonoids (Baba et al. 2009). Consuming a cup of rooibos herbal tea prepared for optimal polyphenol extraction could therefore make a notable contribution to daily dietary polyphenolic content and TAC.

#### Rooibos herbal tea and its health-promoting effects

The fact that rooibos herbal tea is free of caffeine and has a low tannin level in relation to *Camellia sinensis* tea variations had in the past conveyed its ‘health message’ to consumers. Currently, it is its antioxidant capacity, as well as its flavour, that enjoys attention (Joubert et al. 2011). The unique phenolic content of rooibos herbal tea, which, along with other attributes, acts as a potent antioxidant, may be the link to its health-promoting effects (Marnewick, Gelderblom & Joubert 2000). Though experimental research is accumulating to support the anecdotal health-promoting effects of rooibos herbal tea (Marnewick et al. 2011), it is its antimutagenic, anticarcinogenic (Sissing et al. 2011) and cardioprotective (Marnewick et al. 2011) effects that are of particular importance, as cancer and cardiovascular disease (CVD) are major non-communicable diseases of concern among all population groups in South Africa (SA) (Mayosi et al. 2009). Human intervention trials are particularly important to affirm the health-promoting effects of rooibos herbal tea consumption. In this regard, much support has been gained for its cardioprotective effects. Marnewick et al. (2011) evaluated the effect of rooibos on biochemical and oxidative stress parameters in adults at risk for CVD. The study was the first to report the influence rooibos has on the oxidative stress status and the lipid profile of adults at risk for the onset of CVD, which were found to be improved by the daily consumption of six cups of rooibos for 6 weeks. Villano et al. (2010) reported the lipid profile modulation and oxidative stress reduction properties of an acute dose of rooibos (500 mL) in healthy humans.

#### Effects of type, form, preparation method and household flavourings as variables on the total phenolic content and the total antioxidant capacity of rooibos herbal tea

As limited information could be established regarding the above, the effects of these preparation aspects on tea are in the main presented. Bramati, Aquilano and Pietta (2003) quantified the major flavonoids in unfermented rooibos herbal tea. The level of aspalathin, which is one of the main flavonoids in rooibos (Joubert & Schulz 2006), was found to be 50 times higher in unfermented rooibos than in fermented rooibos and the TAC of the unfermented infusion double that of the fermented infusion. Marnewick et al. (2000) and Joubert and Schulz (2006), through their research, confirmed that fermentation decreases the rooibos flavonoid and total polyphenol content.

The form of the tea, along with its weight, are important factors that determine the flavonoid content of steeped tea (Peterson et al. 2004). Ryan and Petit (2010) indicated that tea infused using tea leaves delivered a higher TAC compared to tea infusions prepared from teabags. Cheong et al. (2005) indicated that the flavonoid extraction rate of green tea in powder form was found to be faster than that of green tea leaves. Joubert et al. (2008) furthermore identified low levels of isovitexin, orientin and iso-orientin in powdered rooibos extract produced from rooibos waste materials.

Peterson et al. (2004) determined the influence of preparation techniques on the contents of black tea and reported that it influenced the flavonol content. The flavonoid content of the prepared tea was found to be higher if the steeping time is 4 min or longer, as opposed to shorter steeping times (2 min). Peterson et al. (2004) additionally confirmed that consumers do not infuse their teabags for long enough for a more complete compound withdrawal.

Consumers usually add a sweetener like sugar or honey and/or milk to traditional fermented rooibos herbal tea. Studies performed by Leenen et al. (2000) and Catterall et al. (2003) confirmed that milk addition to tea does not reduce the bioavailability of the antioxidants present in the tea. Phillips, Carlsen and Blomhoff (2009) reported that refined sugars, such as white and brown granulated sugar, have minimal antioxidant activity. It is therefore not expected that these flavourants, on addition, would have an influence on the antioxidant properties of teas. Toydemir et al. (2015) evaluated the effect of the addition of honey on the antioxidant properties of different herbal teas. Their findings showed that the total phenolic content and the TAC of the tea samples with added honey were higher than those without.

#### Consumer tea drinking profile, behaviour and preferences

De Godoy et al. (2013) used a questionnaire to assess consumers’ taste preferences, consumption, drinking behaviour and beliefs regarding Brazilian mate tea. On the question regarding the preferred type of mate tea consumed, the majority of the participants indicated using teabags, with only 5% indicating the use of the loose tea leaf form and 2% instant tea. Most of the participants preferred the temperature of the mate tea to be hot. Most participants indicated consuming tea before bedtime.

Geleijnse et al. (1999) commented that in Western populations, tea consumption is mostly linked with a healthier diet and lifestyle. Their study, which investigated the link between tea consumption and aortic atherosclerosis, found that tea consumption was associated with leaner, better educated people and those who take vitamin antioxidants. The Saudi Arabia National Study (Hakim et al. 2003) indicated that compared to those who consumed less tea (one to three cups and four to six cups daily), the heavy tea consumers (more than six cups daily) were more likely to be younger, male, current smokers, coffee consumers and those having a lower dietary fat intake and a lower treatment rate for blood pressure and diabetes mellitus.

### Research problem statement

The different types and forms in which rooibos herbal tea are available and the ways in which rooibos herbal tea is prepared and flavoured for consumption will influence the total polyphenol content and the TAC of this beverage. Preparation methods of rooibos herbal tea are done according to the specific taste of the consumer. The optimal cup of rooibos herbal tea delivering the highest total polyphenol content and TAC might not necessarily be the most common way consumers drink this beverage.

### Research objectives

The goal of this study was to denote an optimal cup of rooibos herbal tea as having the highest total polyphenol content and TAC, taking into consideration the different rooibos types, forms, preparation methods and the type and amounts of different added household flavourings.

This study further aimed to determine the demographic, lifestyle and rooibos consumption and preparation characteristics of adult rooibos herbal tea consumers in the George area in the Western Cape, SA, and the association of these consumer characteristics with drinking the optimal cup of rooibos.

## Research design and methods

The methodology consisted of two phases: Phase 1 dealt with the selection and preparation of rooibos herbal tea samples. These samples included different rooibos herbal tea types, forms, steeping times and household flavourings and their added amounts for the analysis of the total polyphenol content and the TAC. This provided the composition information for a database to screen the rooibos herbal tea samples for identification of those with the highest polyphenol content and TAC. This was carried out to denote the ‘optimal’ cup of rooibos.

Phase 2 obtained information on how consumers drink their rooibos herbal tea (bearing in mind the identified rooibos beverage with the highest total polyphenol content and TAC in Phase 1), as well as identifying the demographic and lifestyle information of rooibos herbal tea consumers, and in particular those who consume rooibos as a prepared beverage providing the highest total polyphenol content and TAC.

### Data collection procedure for Phase 1

A total of 640 samples were prepared from the different sample brands (two to three brands per rooibos type and form) bought from two different large retail chain supermarkets that offered a wide product range, providing for duplicate samples and prepared to represent each of the various factors investigated. A retail chain supermarket located in the central and in the northern regional sectors of the city of Cape Town was used for this purpose.

A batch for each brand of traditional (fermented) rooibos from each supermarket was made up. Seven teabags from each brand were steeped in 1.26 L of boiling water (to represent one teabag per 180 mL, as the tea volume in a cup) (Langenhoven et al. 1991), stirred for 30 s and steeped for 3 min, which is the steeping time according to the specifications on the Rooibos Limited website (Rooibos, Perfect cuppa 2018).

For all the samples, except for the analysis of the form and type of rooibos herbal tea, traditional fermented rooibos herbal teabags were used, as this form is the most widely available in supermarkets and thus represents the most commonly available rooibos herbal tea form to the rooibos herbal tea consumer. Prepared samples were stored in a test tube at 4°C for analysis and coded for identification purposes.

#### Tea type sample preparations

The different brands of traditional (fermented) (*n* = 5), green (unfermented) (*n* = 1) and organic traditional (fermented) (*n* = 4) rooibos herbal teas purchased were prepared using teabags. The samples were stirred for 30 s and steeped for 3 min according to the specifications on the Rooibos Limited website (Rooibos, Perfect cuppa 2018).

#### Tea form sample preparations

Samples were also prepared from traditional fermented (*n* = 1 and *n* = 1, respectively), green unfermented (*n* = 1 and *n* = 0, respectively) and organic (*n* = 1 and *n* = 0, respectively) rooibos herbal tea leaves and powder. The amount of tea leaves (2.6 g) was weighed and 180 mL of boiling water was added, stirred for 30 s and steeped for 3 min. This amount of tea leaves represents the average weight of tea leaves contained in a teabag.

Only one brand of rooibos herbal tea powder could be prepared (done in triplicate) as this form of tea is not yet readily available on supermarket shelves. A small amount of powder (50 mg) was dissolved in 25 mL of boiling water according to the manufacturer’s preparation instructions. The sample was stirred for 30 s and left for 3 min before the sampling took place.

#### Steeping time sample preparations

Tea samples (*n* = 24) utilising different steeping times were prepared from traditional fermented rooibos herbal teabags. The standard steeping time according to the instructions of the SA Rooibos Council is 3 min. Three different steeping times were applied in relation to the standard steeping time to evaluate different preparation methods. These were chosen to represent quick (1 min), average (5 min) and long (10 min) steeping times (Campanella, Bonanni & Tomasetti 2003). Samples with steeping times of 20 and 30 min were also prepared to determine the effect of a prolonged steeping time on the total polyphenol content and TAC, respectively.

#### Tea sample preparations with additions

The different amounts of household flavourings added were predetermined and are presented in Table 1. Samples were obtained from the prepared tea batches described in the section ‘Data collection procedure for Phase 1’, whereafter each household flavouring was added in the different amounts. Samples with combined household flavourings added and with a different amount of additions per milk type and sweetening agent were also prepared.

**TABLE 1:** Types and amounts and their amount representations of the household flavouring additions.

Household flavouring†	Amount addition			Reference
	Small	Medium	Large	
**Milk, long life**‡				Langenhoven et al. 1991
Skim	10 mL	20 mL	30 mL	
Low fat	10 mL	20 mL	30 mL	
Whole	10 mL	20 mL	30 mL	
**Sugar, granulated**‡				Langenhoven et al. 1991
White	4 g	6 g	10 g	
Brown	4 g	6 g	10 g	
**Honey**‡	5 g	9 g	15 g	Langenhoven et al. 1991

† , the different household flavourings in the different amounts were added to 180 mL (a cup) of rooibos herbal tea.

‡ , for logistical reasons, long life milk cartons were purchased. One batch of milk, sugar and honey were used to eliminate any interference through nuisance variables.

#### Assays

Each 100 microlitre (µL) sample was diluted with 900 µL of distilled water before the assays were performed. Each assay was done in triplicate for precision. All the sample values for a specific factor across the different brand and supermarket purchases were used to calculate an average for that factor. The Trolox equivalent antioxidant capacity (TEAC) (Re et al. 1999; Floegel et al. 2011) and ferric-reducing antioxidant power (FRAP) (Guo et al. 2003) assays were performed to test for the TAC of the samples, while the Folin–Ciocalteau method (Singleton & Rossi 1965) was used for determining the total polyphenol content. For all the assays, the concentration (*x*) of the samples was calculated using the equation *y* = *mx* + *c* of the line on the standard curve where *m* = slope, *c* = *y*-intercept and *y* = absorbance of sample.

#### Data analysis

The SPSS (IBM, Armonk, NY) 22 Statistics Data Editor program was used. One-way analysis of variance (ANOVA) was performed to test for differences between the rooibos herbal tea samples. The dependent variables were the total polyphenol content and the TAC, while the different rooibos types, forms and preparation methods and the added household flavouring types and amounts were the independent variables. Levene’s test for equality of variances and the Student–Newman–Keuls test were applied. Levene’s test for equality of variances as inferential statistic was used to assess the equality of variances for a variable calculated for two or more groups, and the Student–Newman–Keuls Test was conducted as a post hoc test if a positive ANOVA result (*p* < 0.05) was obtained. The Student–Newman–Keuls test through multiple pairwise comparisons determined where the significant (*p* < 0.05) differences in the sample means transpired. The Kruskal–Wallis one-way ANOVA by ranks was performed to determine whether the rooibos samples, where the sample data did not represent a normal distribution, originated from the same population. A positive ANOVA result (*p* < 0.05) obtained was followed by a post hoc analysis through multiple pairwise sample comparisons to determine where the significance (*p* < 0.05) transpired.

### Data collection procedure for Phase 2

#### Questionnaire as data collection tool

Section A of the questionnaire included multiple-choice questions related to the respondents’ beverage, tea and herbal tea consumption. To determine whether a respondent consumed an optimal cup of rooibos herbal tea, the time of infusion was considered. If the respondent chose a steeping time of 10 min or longer it was considered to be an optimal cup of rooibos herbal tea. This was based on the findings of Phase 1.

Section B of the questionnaire consisted of questions related to respondents’ demographics, lifestyle and health, also presented in multiple-choice format. The age category listing started at 25 years of age because of the finding that tea consumers are mostly older persons (Song & Chun 2008). Age was as a result considered an inclusion or exclusion criterion for study participation.

The compiled questionnaire was prescreened for content-related evidence of validity by food and food science (*n* = 3) as well as biochemical (*n* = 2) experts. These experts also considered consumer understanding of the questionnaire (face-related evidence of validity) in their evaluation of the questions. The questionnaire was pilot tested on approximately 10% (*n* = 38) of the envisaged sample size with the respondents representative of the study sample and further amended based on their conveyed comments.

#### Recruitment of respondents and sample formation

A convenient and purposive sample of adult respondents aged 25 years and older who consume rooibos herbal tea and reside in the George area, Western Cape, SA, was recruited through the use of 12 trained recruiters. The distribution of the population by age for the George Municipality from the *Census 2011* (Western Cape) report (Lehola 2012) was used to determine the total number of respondents to be used, as well as the number in each age group, representing the formation of a non-probability quota sample. The sample size was determined by using a margin of error (or precision) of 0.06, a confidence interval of 95% under the normal distribution (i.e. the distribution of when *n* is sufficiently large) and a prior proportion of 0.5.

A minimum sample size of 267 individuals was calculated, but considering the possibility of non-response bias across the age stratus representing the quota sample, the age strata was multiplied by 1.5 to make sure that the required sample size within each stratus was obtained, but having the same ratio representation as the age strata of the population. A total of 344 respondents who met the inclusion criteria were invited to participate, of whom 36 declined, resulting in a response rate of 89.5%. The sample consequently comprised 308 respondents.

#### Data analysis

Frequencies of the questionnaire responses and associations (Pearson’s chi-squared statistic) between the respondents’ consumption of the optimal cup of rooibos herbal tea or not and the respondent profile responses at a significance level of 5% (*p* < 0.05) were used for this data analysis. To account for empty cells and low cell numbers of response options to questions in the questionnaire, some response options were combined to reduce the occurrence of empty cells and low cell counts in the statistical analysis.

### Ethical considerations

Ethical approval for Phase 2 of the study was granted by the Cape Peninsula University of Technology (Ref. 12/2013). All the respondents who participated voluntarily in the study were required to sign the consent form. A participant code was allocated to each respondent to facilitate anonymous participation.

## Results and discussion

### Optimal rooibos cup identification providing the highest total polyphenol content and total antioxidant capacity

The rooibos preparation method across the biochemical parameter analysis providing the highest total polyphenol content and TAC is presented in Table 2. This included the rooibos types and forms available to the consumer and the different steeping times and added household flavourings that were used. The total polyphenol content of the rooibos sample with honey added in a large amount (signifying the household flavouring types and amounts) was significantly (*p <* 0.05) lower compared to that of the sample prepared with powdered rooibos extract (traditional fermented) (signifying the rooibos form) and the sample steeped for 30 min (signifying the rooibos preparation). The powdered rooibos extract (traditional fermented) had a significantly (*p* < 0.05) higher FRAP compared to that of the sample prepared with green unfermented rooibos (signifying the rooibos type) and the sample with the large amount of honey added. The FRAP of the sample prepared with green unfermented rooibos and the sample steeped for 30 min were both significantly (*p* < 0.05) higher than that of the sample with honey added in a large amount. The sample with the large amount of honey added and the iced tea sample (with rooibos extract – traditional fermented) (also signifying the rooibos form) had the highest TAC (measured by the TEAC) with the TAC of these samples also significantly (*p* < 0.05) higher than that of the sample prepared with traditional fermented rooibos (also signifying the rooibos type) and the sample steeped for 30 min.

**TABLE 2:** Rooibos preparation methods providing the highest total polyphenol content and total antioxidant capacity.

Rooibos preparation method	Biochemical content†			Comment
	Total polyphenols‡ (mg/L)	FRAP§ (µmol/L)	TEAC¶ (µmol/L)	
**Rooibos type**††				
Green, unfermented (1)	457.7 ± 40.9‡‡	3219 ± 82§§	-	Limited consumer availability
Traditional, fermented (2)	-	-	1140 ± 143‡‡	-
**Rooibos form**¶¶				
Powdered extract (traditional/fermented) (3)	604.6 ± 39.0‡‡	4004 ± 395‡‡	-	Limited consumer availability
Iced tea (with rooibos extract – traditional/fermented) (4)	-	-	1701 ± 565‡‡	More costly to the consumer, although convenient
Rooibos preparation (brewing time)*				
30 min (5)	543.2 ± 125.7§§	3107 ± 731§§	1212 ± 270§§	-
Household flavouring types and amounts**				
Honey (6)	413.0 ± 72.7§§ (large amount addition as 15 g)	2489 ± 437‡‡ (small amount addition as 5 g)	1868 ± 172§§ (small amount addition as 5 g)	Health implications of use of a large amount of honey (fructose) questionable
Whole milk	519.1 ± 82.7‡‡ (large amount addition as 30 mL)	2100 ± 453‡‡ (large amount addition as 30 mL)	-	High polyphenol content probably because of interference with the absorbance of the assay by some of the milk constituents
Skim milk	-	-	3025 ± 325‡‡ (large amount addition as 30 mL)	-
30 mL whole milk and 15 g honey	545.1 ± 65.7‡‡ (large amount additions as 30 mL and 15 g, respectively)	-	-	High polyphenol content probably because of interference with the absorbance of the assay by some of the milk constituents
30 mL whole milk and 10 g brown sugar	-	2310 ± 609‡‡ (large amount additions as 30 mL and 10 g, respectively)	-	High polyphenol content probably because of interference with the absorbance of the assay by some of the milk constituents
30 mL skim milk and 5 g honey	-	-	3773 ± 146§§ (large and small amount additions as 30 mL and 5 g, respectively)	-
**Significant difference**	(6)-(3)(5)***	(3)-(1)***	(2)-(4)(6)****	-
		(6)-(1)(3)(5)***	(5)-(4)(6)****	

† , biochemical content values: Mean ± standard deviation.

‡ , total polyphenol content presented as milligram (mg) per litre (L).

§ , FRAP: Ferric-reducing antioxidant power assay presented as micromole (µmol) per L.

¶ , TEAC: Trolox equivalent antioxidant capacity assay presented as µmol per L.

†† , rooibos types included traditional (fermented), green (unfermented), organic traditional (fermented).

‡‡ , highest in preparation method grouping.

§§ , significantly highest in preparation method grouping.

¶¶ , rooibos forms included traditional fermented rooibos leaves, green unfermented rooibos leaves, organic rooibos tea leaves, rooibos powder, ready-to-drink unflavoured iced teas with traditional fermented rooibos.

* , steeping times include a quick steeping time (1 min), an average (5 min) and a long (10 min) steeping time (Campanella et al. 2003). Steeping times of 20 and 30 min were also tested to determine the effect of prolonged steeping.

** , the rooibos samples representing the type of milk added and the combined household flavourings were not included in the statistics analysis carried out because of the interference with the polyphenol content analysis of the former (F. Rautenbach, pers. Commun., 10 March 2016).

*** , overall significant (*p* < 0.05) difference in the Levene’s test for equality of variances of the samples with the significant (*p* < 0.05) differences transpiring in the pairwise multiple comparisons of the Student–Newman–Keuls test indicated as a rooibos sample providing a high total polyphenol and total antioxidant capacity (number presented in brackets [e.g. green, unfermented as 1] significantly different (-) to another rooibos sample providing a high total polyphenol content and total antioxidant capacity (TAC) with each sample presented as a number in brackets [e.g. traditional, fermented as 2]).

**** , overall significant (*p* < 0.05) difference in the Kruskal–Wallis Test one-way analysis of variance by ranks of the samples with the significant (*p* < 0.05) contrasts transpiring on the post hoc pairwise multiple comparisons indicated as a rooibos sample providing a high total polyphenol content and total antioxidant capacity (number presented in brackets [e.g. green, unfermented as 1] significantly different (-) to another rooibos sample providing a high total polyphenol content and TAC with each sample presented as a number in brackets [e.g. traditional, fermented as 2]).

Because of limited availability and cost implications to the consumer, the rooibos type, as green unfermented rooibos, and the rooibos form, as powdered rooibos extract (traditional fermented) and iced tea (with rooibos extract – traditional fermented), were not considered to conclude an optimal cup. The sample with the addition of honey in a large amount was also not considered because of the possible health implications of the addition of the large amount of fructose contained in honey. Though there is reason to believe that moderate fructose ingestion could be beneficial for health, an excess intake would be a risk (Livesey 2009). Kretowicz et al. (2011) reported that fructose consumed in excessive amounts might contribute to diabetes and cardiorenal disease as well as obesity and contribute to the development of fatty liver disease (Abdelmalek et al. 2010). Striving for the consumption of at least the four (Yang & Hong 2013) to six (Popkin et al. 2006; Marnewick et al. 2011) cups of tea or rooibos per day that is advised to obtain the health benefits of tea, with a large amount of honey added to each cup, may in view of these health risks be ill-advised.

Permitting for the household preparation of rooibos and taking cognisance of the consumer availability and cost implications of the rooibos type and form, the traditional fermented rooibos in bag form steeped for 30 min was considered as the preparation method that provided for the highest total polyphenol content and TAC. Sharpe et al. (2016) stated that steeping time significantly affects the tea antioxidant capacity. Yet, as the biochemical parameters of the samples steeped for 10, 20 and 30 min did not differ significantly (*p* > 0.05), traditional fermented rooibos in bag form steeped for 10 min or longer was considered to be the optimal rooibos consumption representation to provide for the highest total polyphenol content and TAC. Ryan and Petit (2010) indicated that the antioxidant potential of black tea increased gradually according to the infusion time, with a maximum value obtained with a 10 min steeping time, while Cheong et al. (2005) confirmed that a 10 min steeping time was sufficient for contributing to flavonoid intake.

### Phase 2: Respondent profile and tea drinking behaviour

The sample had a high representation of female (68.2%) and white (70.1%) respondents. The sample furthermore had a near equal representation of respondents aged 25 to 39 years (young adults) (47.4%) and those aged 40 years and older (52.6%) (see Table 3), which represented the envisaged sample age distribution coverage.

**TABLE 3:** Respondent profile and rooibos herbal tea drinking behaviour.

Variable	Characteristics	Total respondent sample (*n* = 308)		Respondents consuming optimal cup of rooibos herbal tea†				Significance (*p* < 0.05)‡
		Frequency (*n*)	Percentage (%)	Yes (*n* = 49)		No (*n* = 259)		
				Frequency (*n*)	Percentage (%)	Frequency (*n*)	Percentage (%)	
**Respondent demographic characteristics**								
Gender	Males	98	31.8	17	34.7	81	31.3	0.620§
	Females	210	68.2	32	65.3	178	68.7	
Age group¶	Young adults	146	47.4	24	49.0	122	47.1	0.735
	Middle aged adults	126	40.9	18	36.7	108	41.7	
	Older adults	36	11.7	7	14.3	29	11.2	
Population group	Black/Indian or Coloured††	92	29.9	15	30.6	77	29.7	0.901
	White	216	70.1	34	69.4	182	70.3	
**Respondent lifestyle and health characteristics**								
Perceived food and beverage intake	Consumed foods/beverages popular with and consumed by most adults of same age	192	62.3	34	69.4	158	61.0	0.334§
	Consumed foods/beverages considered healthier choices	116	37.7	15	30.6	101	39.0	
Physically active	Yes‡‡	158	51.3	23	46.9	135	52.1	0.535§
	No	150	48.7	26	53.1	124	47.9	
Dietary supplement§§ use	Never	108	35.1	19	38.8	89	34.3	0.388
	Seldom	108	35.1	13	26.5	95	36.7	
	Irregular (when remembered, fairly regularly), regularly††	92	29.9	17	34.7	75	29.0	
Smoking status¶¶	Non-smoker	226	73.4	35	71.4	191	73.7	0.727§
	Current smoker/former smoker††	82	26.6	14	28.6	68	26.3	
Perceived body weight status	Underweight/optimal/normal body weight††	185	60.1	28	57.1	157	60.6	0.649
	Slightly overweight/overweight/obese††	123	39.9	21	42.9	102	39.4	
**Respondent rooibos consumption and preparation**								
Consumption frequency	Seldom (almost never, 1–3 cups per month)††	96	31.2	16	32.7	80	30.9	0.968
	Weekly (1–6 cups per week)††	97	31.5	16	32.7	81	31.3	
	One cup per day	37	12.0	6	12.2	31	12.0	
	More than one cup per day††	78	25.3	11	22.4	67	25.9	
Form usually	Tea bags	286	92.9	45	91.8	241	93.0	0.762
consumed	Loose tea leaves/iced tea (ready-to-drink, powder form)††	22	7.1	4	8.2	18	6.9	
Usual preparation method†††	Tea bag steeped in cup or mug	271	88.0	35	74.5	236	91.1	0.001
	Tea bags steeped in teapot	35	11.4	12	25.5	23	8.9	
Temperature at which usually consumed	Hot/near boiling	107	34.7	19	38.8	88	34.0	0.485
	Warm/still heated	184	59.7	26	53.1	158	61.0	
	Cooled/near cold	17	5.5	4	8.2	13	5.0	
Flavouring usually added	Do not add flavouring(s) / lemon juice only††	52	16.9	13	26.5	39	15.1	0.228
	Sweetening agent only	59	19.2	9	18.4	50	19.3	
	Milk only	13	4.2	1	2.0	12	4.6	
	Sweetening agent and milk	184	59.7	26	53.1	158	61.0	

† , usually consumed rooibos strength (represented by steeping time) used as criteria to group the respondents into those consuming an optimal cup of rooibos (*n* = 49) and those who do not (*n* = 259).

‡ , Pearson’s chi-square.

§ , Fisher’s exact test (done for 2 × 2 tables).

¶ , young adults as the age group 25 to 39 years; middle aged adults as the age group 40 to 64 years; and older adults as the age group 65 years and older (Hamarat et al. 2001).

†† , response options grouped together because of low cell counts.

‡‡ , regular moderate exercise (e.g. walking or cycling) or strenuous exercise (jogging, football and vigorous swimming) for 4 h or more per week (Yusuf et al. 2004).

§§ , a vitamin, mineral, herbal, plant extract, amino acid, metabolite, constitute or extract or a combination of any of these substances (Marriot, 2000).

¶¶ , non-smoker have never smoked; current smoker smoked in the last 12 months or quit in the past year and former smoker quit smoking more than a year ago (Yusuf et al. 2004).

††† , tea leaves as response option omitted because of low respondent usage (*n* = 2).

Most (62.3%) of the respondents indicated that they consumed food and beverages popular with and consumed by most adults of the same age and not food and beverages considered healthier choices, were non-smokers (73.4%), had a perceived optimal or normal body weight or considered themselves underweight (60.1% as 56.8% and 3.2%, respectively) and did not use or seldom used dietary supplements (35.1%, respectively), while slightly more respondents indicated they were physically active (51.3%) rather than not (see Table 3). In other studies performed, tea consumption was associated with a healthier lifestyle, with tea intake higher in lean, educated persons who smoked less and also consumed a relatively healthy diet (Geleijnse et al. 2002; Hakim et al. 2003) and occasionally those of higher social class (Geleijnse et al. 2002). The majority of the respondents furthermore indicated that they were not diagnosed with CVD (92.2%), diabetes mellitus type 2 (94.8%), inflammatory conditions (e.g. arthritis) (85.1%) or cancer (skin, lung, breast, liver or prostate) (97.7%) and most indicated that they did not have a family history of these diseases (63.6%, 69.2%, 66.9% and 59.4%, respectively).

Slightly less than a third of the respondents indicated that they consumed rooibos seldom or weekly (31.2% and 31.5%, respectively), while only a quarter (25.3%) of the respondents indicated a consumption frequency of more than one cup per day (see Table 3). This was a rather disappointing finding, as the respondent group comprised individuals who consumed rooibos herbal tea. Marnewick et al. (2011) reported a consumption of six cups of rooibos daily to reduce the risk for CVD in adults and Joubert and de Beer (2012) advised six cups of rooibos herbal tea as a minimum daily consumption to have a beneficial health effect.

Furthermore, two to three cups of tea or herbal tea per day were indicated by almost a third (32.1%) of the respondent sample as their daily consumption frequency of tea and herbal tea, while a very small number of the respondents indicated a consumption frequency of four to six cups and more than six cups per day (17% and 6%, respectively). This was a further disappointing finding. Popkin et al. (2006) advised the consumption of five to six cups and Yang and Hong (2013) four to six cups of tea on a daily basis to be beneficial for human health.

The majority of the respondents usually used rooibos in the form of teabags (92.9%). Although the majority (88%) of the respondents indicated that they steeped their teabag in a cup or mug, and only 11.4% of the respondents indicated that they usually prepared their teabags steeped in a teapot; this delivered a significant (*p* < 0.05) difference in the preparation method of those consuming an optimal cup of rooibos and those who did not (see Table 3). It was these latter respondents who were inclined to consume an optimal cup of rooibos. This was the only significant difference to be identified between those respondents consuming an optimal cup of rooibos and those who did not across the respondent demographic, lifestyle and health characteristics, as well as their rooibos consumption and preparation traits (see Table 3). Just over a quarter (26.9%) of the respondents indicated that they consumed their rooibos at weak to medium strength (steeped between 1 and 5 min), while a quarter (25%) indicated that they consumed it at medium strength (steeped for 5 min). Less than a fifth (15.9%) of the respondents indicated that they usually consumed rooibos that had been steeped for 10 min (7.5% or 23 respondents) or longer than 10 min (8.4% or 26 respondents). A mere 15.9% of the respondents hence consumed an optimal cup of rooibos, based on a steeping time of 10 min or longer, which was set as the criteria to group the respondents into those consuming an optimal cup of rooibos (*n* = 49) and those who did not (*n* = 259) (see Table 3).

More than half (59.7%) of the respondents indicated that they consumed rooibos warm or still heated, while about a third (34.7%) indicated that they consumed it hot or near boiling. A sweetening agent together with milk was indicated by more than half (59.7%) of the respondents as their additions to rooibos, while only a fifth (19.2%) of the respondents indicated that they only added a sweetening agent and only 13 respondents (4.2%) that they only added milk (see Table 3). Just more than a fifth (23.7%) of the respondents indicated that they usually added white sugar and whole milk, which was followed by about a tenth (11.4%) of the respondents who indicated that they usually added white sugar and low fat milk.

## Limitations

The interference of the non-phenolic components present in milk in the assays resulted in a false total polyphenol content, hence not providing an accurate value for the samples with milk added as flavouring. The market unavailability of some of the types and forms of rooibos does not make the biochemical parameter findings related to these samples of pertinent value to the consumer. As the respondent selection for the quota sample was non-random, sample bias is an almost certain limitation of Phase 2 of the study. The sample would also have been more representative if more individuals from the non-white population groups completed the questionnaire. The few research studies completed that have a likeness to that of Phase 1 (Catterall et al. 2003; Cheong et al. 2005; Leenen et al. 2000; Peterson et al. 2004; Phillips et al. 2009; Toydemir et al. 2015) and those similar to Phase 2 (De Godoy et al. 2013; Geleijnse et al. 1999; Hakim et al. 2003) of this study have mostly been conducted on other types of tea and in other countries. This restricted the discussion of the results of this study.

## Recommendations

By addressing the daily amount of rooibos consumed and applying a longer steeping time, the consumption of rooibos herbal tea can make a valuable contribution to the daily dietary TAC. Van Graan et al. (2013) described the importance of consuming lots of clean, safe water as part of the South African food-based dietary guidelines. The general recommendation for total daily water intake is between 2 L and 3.7 L for adult women and men. Water is an important liquid nutrient as it is involved in important functions of the body and it is also the key dietary source of fluoride. The dietary sources of fluid include drinking water and other beverages and foods that contain water (Van Graan et al. 2013). The intake of tea can make an important contribution to the water or fluid intake, as well as fluoride intake, of an individual and should be encouraged. Rooibos herbal tea as a beverage is mostly made up of water and additionally provides protective effects against disease occurrence, in particular those associated with oxidative stress (Baba et al. 2009), which is ascribed to the phenolic constituents it contains (Van Heerden et al. 2003). The rooibos industry should strive to increase the consumption of rooibos by its consumers and inform them that a longer steeping time provides for a more optimal cup in terms of the total polyphenol content and the TAC.

## Conclusions

The different rooibos types, forms and preparation methods in particular had an influence on the total polyphenol content and TAC of rooibos consumed as a beverage. The rooibos sample steeped for 10 min or longer was identified as the optimal cup of rooibos. A mere 15.9% of respondents consumed an optimal cup of rooibos. There was no significant difference between the respondents consuming an optimal cup of rooibos and those who did not, where their demographic and lifestyle characteristics were concerned. The only significant difference found was related to the preparation method of rooibos. Although the respondents generally steeped their teabag in a cup or mug, more of those respondents who steeped it in a teapot consumed the optimal cup considering the steeping time of 10 min and longer. This finding should be considered in relation to the finding that these respondents as rooibos consumers also did not consume it in an amount (as a minimum of 4 to 6 cups per day) that would fully provide for obtaining its health benefits, while a very small number of the respondents indicated a consumption frequency of four to six cups and more than six cups of tea or herbal tea per day. Rooibos herbal tea was hence not consumed enough by the respondents, as only a few indicated that they consumed four or more cups per day. Concerning the demographic and lifestyle characteristics of these rooibos herbal tea consumers, it can be concluded that they can generally be assumed to be healthy and practising healthy lifestyle behaviours.
